# Special Issue “Viral Infections and Host Immune Responses”

**DOI:** 10.3390/ijms262110382

**Published:** 2025-10-25

**Authors:** Maria Teresa Maggiorella, Barbara Ridolfi

**Affiliations:** 1 National HIV/AIDS Research Center, Istituto Superiore di Sanità, Viale Regina Elena 299, 00161 Rome, Italy; mariateresa.maggiorella@iss.it; 2 National Center for Global Health, Istituto Superiore di Sanità, Viale Regina Elena 299, 00161 Rome, Italy

The emergence of viral epidemics, climate change, and population migration has resulted in greater vulnerability to the transmission of old, new, and re-emerging infectious diseases. To respond effectively to viral infections, the immune system must assess the threat posed by each pathogen by analyzing its molecular components and comparing them to those of the host. In viral infections, the host’s innate immune system is designed to act as the first line of defense to prevent viral invasion or replication before the adaptive immune system generates a more rapid and robust protective response. Some viruses, such as influenza virus and rhinovirus, can stimulate immediate antibody production and an effective immune response in the host. As a result, in most cases, these pathogens are definitively eradicated from the organism after an acute illness phase. This is not the case with other viruses such as HIV, HCV, and Herpesviruses, which often lead to latent infections and chronic viral diseases. In this Guest Editors’ overview, we discuss four original articles [1,2,3,4] and a review [5], aimed at deepening our understanding of the complex interplay between viral pathogens and the host immune system. This collection features novel research that highlights recent advances and emerging applications in the field of “Viral Infections and Host Immune Responses”.

Coronaviruses were a major research focus during the COVID-2019 pandemic. Despite the development of numerous antiviral strategies and vaccines in record time, much remains to be discovered about the virus’s interaction with host cells and the immune system. In their article, Qudus et al. [1] explore how the SARS-CoV-2 accessory protein ORF3a affects host cell processes, specifically apoptosis and mitochondrial function, which are crucial aspects of the host’s immune response to viral infection [6]. The study demonstrates that in vitro the SARS-CoV-2-ORF3a protein triggers apoptosis by altering the mitochondrial homeostasis and upregulating the expression of the Mitochondrial ATP-sensitive Potassium Channel (MitoK_ATP_). In addition, in an in vivo mouse model, ORF3a transfection triggers significant mitochondrial damage and enhances apoptosis and the inflammatory response in lung tissue. Interestingly, in their experimental models, the authors report that ORF3a induces an increase in interferon-beta (IFN-β) expression levels, a cytokine that plays a pivotal role in the innate immune response to viral infections. Furthermore, they find that the addition of exogenous K+ mitigates both the apoptotic and inflammatory responses induced by SARS-CoV-2-ORF3a. These findings suggest that targeting potassium ion channels may offer a promising therapeutic approach to mitigate the harmful effects of SARS-CoV-2 infection. Moreover, these results highlight the need for a nuanced strategy when developing therapies aimed at ORF3a, as these treatments must carefully balance the protein’s dual functions in promoting apoptosis and triggering the antiviral immune response. Selective inhibitors should therefore minimize viral cellular damage while preserving innate immunity.

The article by Martianez-Vendrell et al. [2] examines the main protease (Mpro) of the human coronavirus HCoV-229E and its role in manipulating the host immune system. Specifically, it explores how the Mpro of this common cold strain interferes with the host immune response by targeting the RIG-1-like receptor (RLR) pathway, which is involved in detecting viral RNAs and triggering antiviral responses. The authors report that the Mpro of HCoV-229E suppresses this RLR-mediated signaling, preventing the activation of key antiviral defenses. Mpro suppresses immune signaling by cleaving the NF-kB essential modulator (NEMO) protein, which is crucial in the activation of NF-kB pathways. Furthermore, the article discusses additional mechanisms employed by the viral M protease to evade or suppress host immune responses. This research shows that immune evasion activities in the Mpros of highly pathogenic coronaviruses are also present in the Mpros of human common cold coronaviruses. These findings underscore the importance of comparative studies between coronaviruses of different pathogenicity to determine the role played by the Mpro in disease severity.

Although combination antiretroviral therapy (cART) is a highly effective treatment for people with HIV, it does not lead to a cure from the infection because it does not affect the HIV-1 latent reservoir. One of the most explored cure approaches is the “shock and kill” strategy, which aims to reactivate the latent HIV-1 proviruses and subsequently kill the virus-producing cells [7]. In this context, but with a new perspective, Vlaming et al. investigated the synergistic effects of combining a latency reversal agent, such as a second mitochondrial-derived activator of caspases mimetic (SMACm), with Toll-like receptor 8 (TLR8) or RIG-I-like receptor (RLR) agonists to enhance both viral reactivation and immune-mediated clearance [3]. Using ex vivo PBMCs from untreated HIV-positive individuals, they showed that the combination of SMACm and the TLR8 agonist resulted in an additional 25% reduction in the reservoir compared to SMACm alone. Although the increase in reservoir reduction was small, interestingly, they observed that co-stimulation elicited a strong adaptive immune response. These findings provide proof of concept for a dual-targeted approach to HIV-1 reservoir elimination. Clearly, further validation of efficacy and safety in different patient cohorts is required for clinical translation. Research in this field, particularly on SMACm-related models or similar immune modulators, could pave the way for new therapeutic strategies.

Infection by oncogenic human papillomavirus (HPV) is the primary cause of cervical cancer and several other anogenital cancers. Enhancing the immune system’s ability to recognize HPV could, therefore, help prevent or control infection. Surfactant proteins (SP), particularly SP-A and SP-D, are key components of the innate immune system that facilitate the detection and clearance of pathogen recognition and phagocytosis through opsonization [8]. Although first discovered in the lungs—where they contribute to pulmonary surfactant function, homeostasis, and defense against infection—they are also found in other tissues. It is noteworthy that their presence in areas such as the female reproductive tract implies they may have additional, underexplored roles in immune defense outside the lungs [9]. The article by Carse et al. contributes original findings on how host immune molecules (SP-A) can modulate immune responses to HPVs and potentially be harnessed for therapeutic or prophylactic purposes [4]. The authors previously identified the surfactant protein A (SP-A) as a novel molecule capable of recognizing HPV16 pseudovirions (HPV16-PsVs) and reducing infection in a murine cervicovaginal challenge model [10]. In the present study, they demonstrated that SP-A can agglutinate and opsonize multiple oncogenic HPV-PsV types, enhancing their uptake and clearance by murine macrophages and THP-1 human-derived immune cells. The opsonization of HPV with SP-A not only enhanced lysosomal accumulation in macrophages and HaCaT keratinocytes but also reduced the infection rate in HaCaT cells. Further analysis revealed that SP-A enhanced cytokine response to HPV16-PsVs of human innate immune cells. These findings underscore the multifaceted impact of SP-A on HPV infection, innate immune cells, and keratinocytes, thereby establishing a basis for the development of novel therapeutic or prophylactic interventions against a broad spectrum of HPV types.

Herpesvirus infections are clinically important due to their ability to establish latency and potentially reactivation, posing serious risks for immunocompromised individuals, including organ and hematopoietic stem cell transplant recipients [11]. The review by Gomes Torres et al. [5] explores new approaches and advancements in loop-mediated isothermal amplification (LAMP), a nucleic acid amplification technique (NAAT), for virus diagnosis. The review emphasizes the detection challenges posed by Herpesviridae, whose infections—especially during reactivation in transplant and immunocompromised patients—are complex and difficult to manage. By exploring these advancements, the authors provide insights that could potentially contribute to improved management of viral infections, ultimately informing immune response strategies and enhancing patient outcomes in transplantation settings.

In summary, the articles included in this collection explore key research areas in the field of human viral infections and host immune responses. Collectively, these studies yield important insights and highlight emerging avenues that may inform the development of broad-spectrum antiviral strategies and novel therapeutic interventions.
